# Design of Bismuth Tungstate Bi_2_WO_6_ Photocatalyst for Enhanced and Environmentally Friendly Organic Pollutant Degradation

**DOI:** 10.3390/ma17051029

**Published:** 2024-02-23

**Authors:** Aicha El Aouni, Mohamed El Ouardi, Madjid Arab, Mohamed Saadi, Henrik Haspel, Zoltán Kónya, Abdelkader Ben Ali, Amane Jada, Amal BaQais, Hassan Ait Ahsaine

**Affiliations:** 1 Laboratoire de Chimie Appliquée des Matériaux, Centre des Sciences des Matériaux, Faculty of Sciences, Mohammed V University, Rabat 1014, Morocco; aicha.elaouni@um5r.ac.ma (A.E.A.); mohamed.elouardi@um5r.ac.ma (M.E.O.); m.saadi@um5r.ac.ma (M.S.); a.benali@um5r.ac.ma (A.B.A.); 2 Aix Marseille University, Université de Toulon, CNRS, IM2NP, CS CEDEX 9, 60584 Toulon, France; madjid.arab@univ-tln.fr; 3 HUN-REN-SZTE Reaction Kinetics and Surface Chemistry Research Group, Rerrich Béla tér 1, H-6720 Szeged, Hungary; haspel@chem.u-szged.hu (H.H.); konya@chem.u-szged.hu (Z.K.); 4 Department of Applied and Environmental Chemistry, University of Szeged, Rerrich Béla tér 1, H-6720 Szeged, Hungary; 5 Institute of Materials Science of Mulhouse (IS2M), Haute Alsace University, 68100 Mulhouse, France; amane.jada@uha.fr; 6 Strasbourg University, 67081 Strasbourg, France; 7 Department of Chemistry, College of Science, Princess Nourah Bint Abdulrahman University, P.O. Box 84428, Riyadh 11671, Saudi Arabia; aabaqeis@pnu.edu.sa

**Keywords:** bismuth tungstate, photocatalysis, degradation mechanism, active species

## Abstract

In this study, a chemical precipitation approach was adopted to produce a photocatalyst based on bismuth tungstate Bi_2_WO_6_ for enhanced and environmentally friendly organic pollutant degradation. Various tools such as X-ray diffraction (XRD), Raman spectroscopy, scanning electron microscopy (SEM), optical spectroscopy and X-ray photoelectron spectroscopy, were employed to assess the structural and morphological properties. Hence, the XRD profiles showed a well crystallized Bi_2_WO_6_ orthorhombic phase. The photocatalytic performance of the resulting photocatalyst was assessed by the decomposition of Rhodamine B (RhB) and methyl orange (MO) with a decomposition efficiency of 97 and 92%, along with the highest chemical oxygen demand of 82 and 79% during 120 min of illumination, respectively. The principal novelty of the present work is to focus on the changes in the crystalline structure, the morphology, and the optical and the photoelectrochemical characteristics of the Bi_2_WO_6_, by tuning the annealing temperature of the designed photocatalyst. Such physicochemical property changes in the as-prepared photocatalyst will affect in turn its photocatalytic activity toward the organic pollutant decomposition. The photocatalytic mechanism was elaborated based on electrochemical impedance spectroscopy, photocurrent analysis, photoluminescence spectroscopy, and radical trapping measurements. The overall data indicate that the superoxide O_2_^•−^ and holes h^+^ are the principal species responsible for the pollutant photodegradation.

## 1. Introduction

With the global advancement of urbanization, economy, and industry, various domestic activities and industrial discharges are generating a wide range of pollutants. These pollutants have high bioconcentration capacities in the environment, leading to severe atmospheric damage and negative impacts on all life forms [1,2,3,4,5,6,7]. Mainly, the discovery of the photocatalytic phenomenon in 1969 opened up new avenues for effectively decontaminating air, water, and soil through extensive research [8,9,10,11,12,13]. Consequently, significant research efforts have been channeled toward investigating the reaction between complex dye pollutants and various disinfectants. Various advanced oxidation technologies have been engineered to achieve environmentally friendly pollutant removal, including adsorption [14,15,16,17], electrocatalytic degradation [18,19,20], and photodegradation [21,22,23]. The ultimate goal is to degrade these organic contaminants into harmless substances like H_2_O and CO_2_ [24,25,26]. To achieve this, researchers have focused on developing innovative photocatalysis methods, particularly utilizing common photocatalysts with wider energy bandgaps, including oxides with diverse crystalline structures [27]. However, more recent studies have been focused on the creation of new visible-light-induced photocatalysts. Some of the materials currently under investigation include semiconductor oxides like NaTaO_3_ [28], CaIn_2_O_4_ [29], γ-Bi_2_MoO_6_ [30], Bi_4_Ti_3_O_12_ [31], and Bi_2_WO_6_ [32]. These materials offer a promising way to advance the area of photocatalysis and its potential for environmentally friendly pollutant degradation.

Among numerous visible-light-driven photocatalysts, Bismuth tungstate (Bi_2_WO_6_) has garnered considerable interest within the scientific community thanks to its distinctive optical, electronic, and photocatalytic characteristics [33,34]. Its structural composition contains interconnected layers of [Bi_2_O_2_]^2+^ and WO_6_ octahedra [23]. This layered configuration of Bi_2_WO_6_ offers advantages in promoting charge transfer and minimizing the recombination of photo-generated electron-hole charges, which are crucial processes in photocatalysis and other photoactive applications [35,36,37]. Thus, with a band gap of 2.8 eV, Bi_2_WO_6_ exhibits a propensity for absorbing in an extended wavelength region. This results in high efficiency in breaking down pollutants upon exposure to visible light, making it particularly suitable for solar energy-driven environmental remediation, such as water purification and air treatment [18,19]. In order to widen the visible light absorption and enhance the photocatalytic performance of Bi_2_WO_6_, different methods have been proposed. For instance, high-quality Bi_2_WO_6_ nanoparticles (NPs) were obtained via hydrothermal approaches [38], sol-gel procedures [39], microwave synthesis [40], chemical precipitation [41], and solvothermal methods [42].

In the present work, to design Bi_2_WO_6_ nanoparticles (BWO), we have used the traditional chemical precipitation method, due to its simple, inexpensive, and surfactant-free attributes. Thereafter, the as-prepared samples were calcined at 400 °C, 500 °C, and 600 °C in order to investigate the effect of calcination temperature on the structure and properties of the resulting materials. Various analytical techniques including powder X-ray diffraction (XRD), scanning electron microscopy (SEM), Raman spectroscopy, X-ray photoelectron spectroscopy (XPS), UV-vis diffuse reflectance spectroscopy (DRS), and photoluminescence spectroscopy (PL) were employed to assess the structural, morphological, and optical characteristics of the prepared materials. Further, the effects of the designed photocatalyst calcination on its crystalline properties, its morphology, and its pollutant removal efficiencies were systematically examined. The photodegradation performance was evaluated by RhB and MO dye-removing ability in an aqueous medium under irradiation with visible light. Additionally, we investigated the active species engaged in the breakdown of RhB over the Bi_2_WO_6_ and assessed the cycling performance of the catalyst as well.

## 2. Experimental Section

### 2.1. BWO Synthesis

All chemicals employed here were of analytical grade from Sigma-Aldrich, and were processed directly without further purification. Typically, Sodium tungstate dehydrate [Na_2_WO_4_·2H_2_O], bismuth nitrate pentahydrate [Bi(NO_3_)_3_·5H_2_O], nitric acid (HNO_3_), and ammonium hydroxide (NH_4_OH) were used as precursors.

Initially, Bi(NO_3_)_3_·5H_2_O was solubilized in a volume of diluted HNO_3_ (10%, *v*/*v*) solution at 25 °C. The resulting nitrate solution was then dropped into 0.1 L of aqueous Na_2_WO_4_·2H_2_O solution under vigorous stirring for 2 h. The pH was set to pH = 5.5 by introducing NH_4_OH to the solution, which was then placed in a condenser surrounded by an oil bath under continuous agitation. The solution temperature was kept between 70 and 80 °C for 24 h. The obtained white precipitate was isolated by centrifugation, washed repeatedly with distilled water and finally dried in an oven. Part of the as-prepared BWO powder was subjected to calcination in a tube furnace at 400 °C, 500 °C, and 600 °C for 3 h under flowing argon atmosphere.

### 2.2. Material Characterization

The BWO powder was structurally investigated by XRD using an EMPYREAN Panalytical (Toulon University France) diffractometer mounted with a copper X-ray source (wavelength λ = 1.5440 Å) and a Ni filter to eliminate K_β_ radiation. The 2Θ range of 10–80° was scanned with a step size of 0.026°. Raman spectroscopy was employed to characterize the vibrational properties of the obtained materials. The Raman profile was acquired by a JASCO (Toulon University, France) spectrometer operated with a 633 nm laser. The BWO grain morphology was examined by scanning electron microscopy (SEM) (Thermo-Fisher Scientific Apreo C, Szeged, Hungary) equipped with a Röntec QXE energy dispersive X-ray spectrometer. The survey and high-resolution XPS spectra were performed by X-ray photoelectron spectroscopy (XPS) by using a (Mulhouse, France) coupled with a concentric hemi-spherical analyzer. To determine the absorption range and band gap energy of BWO, the absorption profile was recorded in the 200–800 nm wavelength interval using an ultraviolet-visible spectrophotometer (JASCO UV-Vis V-730). Photoluminescence (PL) measurements were registered using a Fluoromax fluorescence spectrophotometer (Horiba, Toulon, France).

### 2.3. Photocatalytic Procedure

A representative photocatalytic RhB and MO decomposition measurement was as follows. A total of 100 mg of BWO was solubilized in 100 mL RhB and MO solutions (5 ppm) and the pH of the suspensions was set to pH = 5.5 and 5.8, respectively. After ultrasonication for 10 min, the resulting suspensions were agitated in the dark for an additional 2 h to achieve adsorption–desorption equilibrium between BWO and the dyes. The mixture was illuminated with a Philips lamp (tungsten lamp 300 W), while being continuously stirred. At specific time intervals, 3 mL of the solution was withdrawn and centrifuged at 13,400 rpm for 10 min to eliminate the BWO from the suspension. The evolutions of MO and RhB decomposition were studied using a Shimadzu (Kyoto, Japan) UV 2600 spectrophotometer by measuring the aqueous solution absorbance in the 400–800 nm wavelength region. To confirm the radicals produced by the BWO under irradiation and responsible for the photocatalytic activity, a series of scavenger tests was conducted. Thus, chemical scavengers such as Ethylene Diamine disodium Tetra-Acetate (EDTA), Isopropanol (IPA), and L-ascorbic acid, were selected to trap the active species h^+^, OH^•^, and O_2_^•−^, respectively.

### 2.4. Measurement of Zero Charge Point

The point of zero charge (pH_pzc_) can be described as the pH where the charges resulting from negative and positive surface groups are equal. The pH_pzc_ of the BWO surface was obtained by the method proposed by Al-Harahsheh [43]. Thus, a sample amount of 0.05 g was transferred to six beakers filled with 50 mL of 0.1 M potassium nitrate (KNO_3_) solution. To obtain the curve of the zero-point charge measurements, the initial pH values of the beakers were set at to 2.2, 4.3, 6.1, 8.2, 10.4, and 12.1 by adding some drops of 0.1 M HNO_3_ or 0.1 M NaOH. The obtained suspensions were left to equilibrate for one day. After filtration, the final pHs were measured.

### 2.5. Photoelectrochemical Measurement

Photoelectrochemical analyses were undertaken using an OrigaLys ElectroChem (Rillieux-La-Pape, France) electrochemical workstation in a three-electrode configuration. Ag/AgCl and platinum foil were chosen as reference and counter electrodes, respectively. Sample-coated indium tin oxide (ITO) substrates were used as working electrodes (WE) in 0.1 M sodium sulfate (Na_2_SO_4_) aqueous electrolyte. The visible light source was a 300 W Xe lamp fitted with a 420 nm cut-off filter (λ > 420 nm). The WE was fabricated on ITO glass by drop casting. In a typical deposition, ITO glass slides were cleaned with acetone and ethanol. To prepare the electrode, a mass of 10 mg of photocatalyst powder was mixed with 2 mL of absolute ethanol by ultra-sonication for 15 min to form a well-dispersed suspension. Afterward, 100 μL of the suspension was dropped onto a 1 × 1 cm^2^ ITO substrate and dried at 100 °C for 5 h. The transient photocurrent was measured under 0.5 V bias. Electrochemical impedance spectra (EIS) were recorded in the 0.01–10^5^ Hz frequency range using an amplitude of 5 mV, while Mott–Schottky graphs were constructed at a frequency of 500 Hz.

### 2.6. Chemical Oxygen Demand (COD)

The mineralization of the cationic dye (RhB) and anionic dye (MO) during photocatalytic reaction has been assessed through measuring the chemical oxygen demand (*COD*). RhB and MO photodecomposition tests were conducted in a 100 mL beaker at ambient temperature. The *COD* was measured by using a Lovibond *COD* kit (Dortmund, Germany), and the results were obtained using an MD 200 *COD* spectrophotometer. The rate of photodecomposition was defined as *COD_t_*/*COD*_0_ vs. time. The rate was computed for different photocatalytic reaction times according to the following equation:

(1) $$COD\left(\%\right)=\left(\frac{{COD}_{0}-{COD}_{t}}{{COD}_{0}}\right)\times 100$$

*COD*_0_: *COD* content (mg/L of O_2_) before decomposition.

*COD_t_*: *COD* content (mg/L of O_2_) at a time t.

## 3. Results and Discussion

### 3.1. Structural Investigation

**Figure 1:**
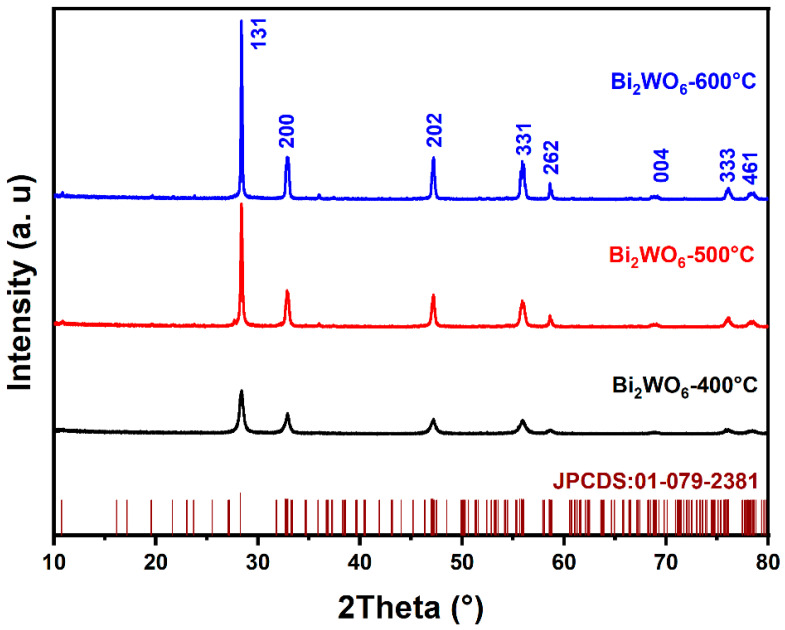
XRD patterns of BWO NPs annealed at 400, 500, and 600 °C.

The diffraction profiles of BWO NPs annealed at 400, 500, and 600 °C are presented in Figure 1. As shown in the figure, all peaks in the XRD profiles are in good conformity with the JCPDS card No. 01-079-2381 and correspond to the pure BWO orthorhombic phase. The determination of grain size involved the application of the Scherer formula, leading to grain sizes of 14, 24, and 28 nm for the BWO NPs after calcination at temperatures of 400, 500, and 600 °C, respectively. Meanwhile, the lattice parameters and unit cell volumes of the BWO NPs after calcination at varying temperatures were determined using the following equations.

(2) $$\frac{1}{d_{hkl}^{2}}=\frac{h^{2}}{a^{2}}+\frac{k^{2}}{b^{2}}+\frac{l^{2}}{C^{2}}$$

(3) V=abc

The results for calcined BWO NPs are shown in Table 1 and reveal a close alignment between the experimental findings and the established reference values for orthorhombic BWO. Moreover, as the treatment temperature increases, differences emerge in the response of the crystal lattice along its distinct axes. These variations can be attributed to the material’s propensity for structural phase transitions at varying temperature levels. Consequently, a significant augmentation in grain size is observed, likely attributable to the process of recrystallization and the aggregation of ultrafine grains. 

### 3.2. Vibrational Spectroscopy

**Figure 2:**
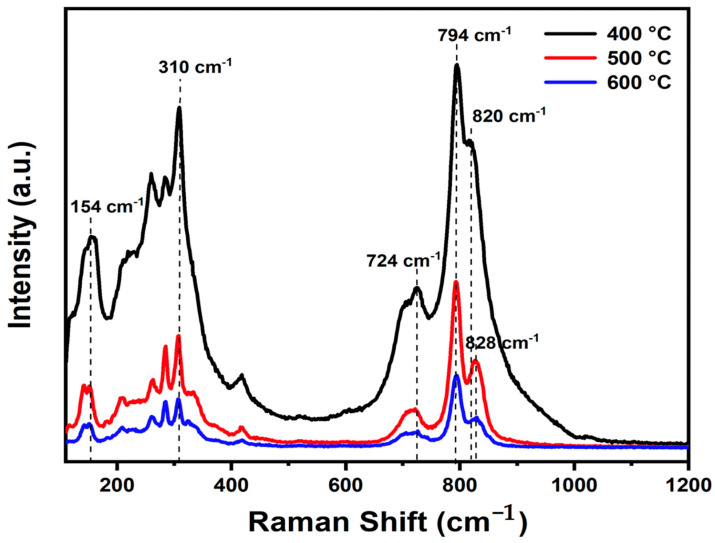
Raman spectra of the BWO NPs calcined at 400, 500, and 600 °C.

The experimental Raman spectra of Bi_2_WO_6_ with characteristic peaks in the wavenumber region of 110 to 1200 cm^−1^ at various temperatures are presented in Figure 2. The BWO400, BWO500, and BWO600 samples show the characteristic Raman peaks of Bi_2_WO_6_. The Raman strong peaks at 818 cm^−1^ and 794 cm^−1^ corresponded to the symmetric and antisymmetric A_g_ modes of terminal O–W–O, respectively. Changes in calcination temperature result in alterations in the shape and position of the O–W–O vibration bands, with the 794 cm^−1^ band shifting to lower wavenumbers and the 818 cm^−1^ band shifting to higher wavenumbers [44]. Additionally, the presence of a peak at 724 cm^−1^ signifies the tungstate chain’s antisymmetric bridging mode, with its wavenumber decreasing as the calcination temperature decreases [45]. Moreover, the peak at 310 cm^−1^ is ascribed to the translation mode due to the simultaneous movements of Bi^3+^ and WO_6_, while the 154 cm^−1^ peak relates to external vibrations of the WO_6_ octahedron. Peaks observed between 200 and 520 cm^−1^ primarily arise from bending vibrations or stretching of the Bi–O bonds, BiO_6_ polyhedron, and WO_6_ octahedron [46].

The peaks are arranged linearly at 820 cm^−1^ and 828 cm^−1^, indicating a clear distortion in bond length with temperature of calcination. The O–W–O bond length of the samples was determined according to the given formula [47].

(4) $$\nu \left({cm}^{-1}\right)=21,349\cdot e^{\left[1.917\cdot R\left(Å\right)\right]}$$

where *R* is the bond length (Å) and *ν* is the Raman stretching frequency (cm^−1^). The computed bond lengths for the studied BWO samples are given in Table 2.

Furthermore, it is noteworthy that the Raman peaks of BWO-500 and BWO-600 show decreased intensity and increased width in comparison to BWO-400. This change in bond length could potentially be ascribed to the recrystallization of BWO NPs at elevated calcination temperatures.

### 3.3. Morphology Analysis

**Figure 3:**
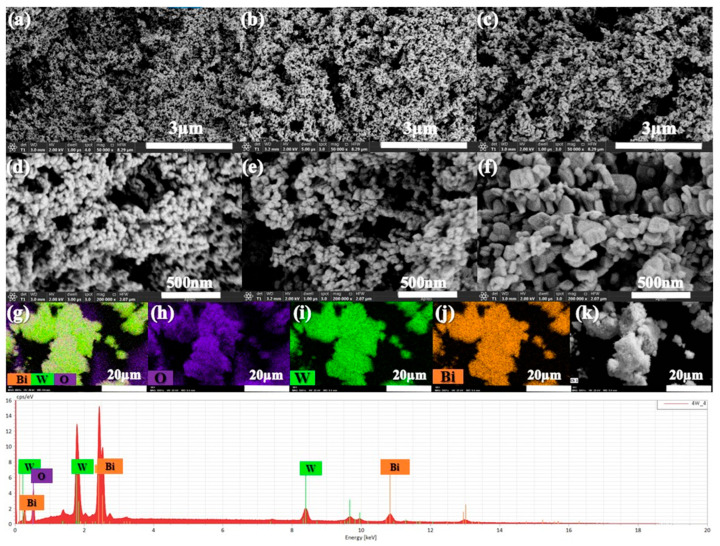
SEM analysis of the BWO NPs synthesized at 400 °C (**a**,**d**), 500 °C (**b**,**e**), 600 °C (**c**,**f**), and the elemental mapping (EDS) images (**g**–**k**) of BWO400.

The scanning electron microscopy (SEM) analyses are presented in Figure 3 and provide insights into the morphology of BWO NPs, enabling the observation of variations in particle size and shape resulting from different calcination temperatures. At all three calcination temperatures, Figure 3a,d correspond to a calcination temperature of 400 °C, Figure 3b,e to 500 °C, and Figure 3c,f to 600 °C. The morphology reveals the presence of uniformly spherical particles. Notably, the size distribution of these particles at 400, 500, and 600 °C measures less than 1 μm. Additionally, it is obvious that the BWO NPs size increases progressively with higher calcination temperatures, aligning well with the findings of X-ray diffraction analysis. This observed rise in particle size can be explained by the agglomeration of nanoparticles that occurs at elevated calcination temperatures. SEM-mapping and EDS spectra were conducted to study the elements’ distribution across the particle surface, as illustrated in Figure 3g–k. The results demonstrate a relatively consistent distribution of O, W, and Bi elements on the surface of the sample particles. These observations align well with the findings from Raman and XRD analyses.

### 3.4. Optical Properties

**Figure 4:**
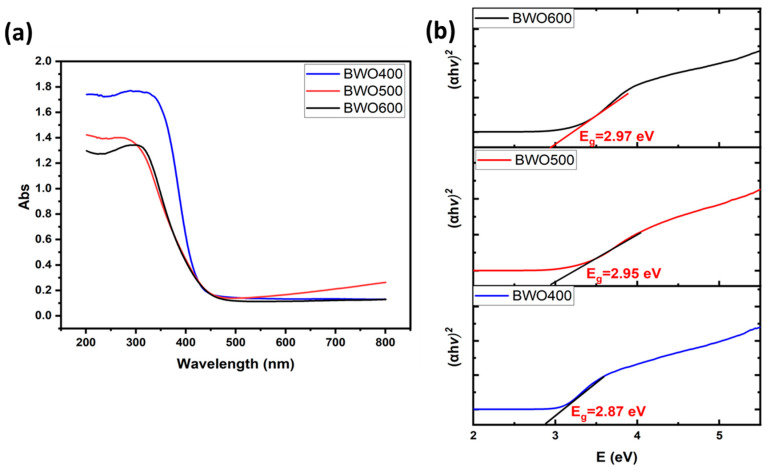
UV-visible diffuse reflectance graph of BWO NPs annealed at 400, 500, 600 °C (**a**), and their corresponding Tauc plots with band gap energies indicated (**b**).

UV-vis diffuse reflectance spectroscopy was employed to explore the annealing temperature effect on the optical characteristics of the catalysts and to derive their bandgap energy (E_g_) values. Figure 4a displays the diffuse reflectance graph of BWO with excellent optical absorption and shows that the absorption of light is enhanced as the annealing temperature decreases. The bandgap energy (E_g_) was determined from Tauc’s equation, (αhν)^1/γ^ = B (hν − E_g_) [48], in which ν: photon frequency, α: absorption coefficient, h: Planck’s constant, and γ: coefficient = 2 or 1/2 for both indirect and direct band gaps. Several investigations have shown that BWO has a direct band gap [49,50].

The resulting E_g_ values were found to be 2.87, 2.95, and 2.97 eV for BWO400, BWO500, and BWO600, respectively (Figure 4b).

### 3.5. X-ray Photoelectron Spectroscopy (XPS)

**Figure 5:**
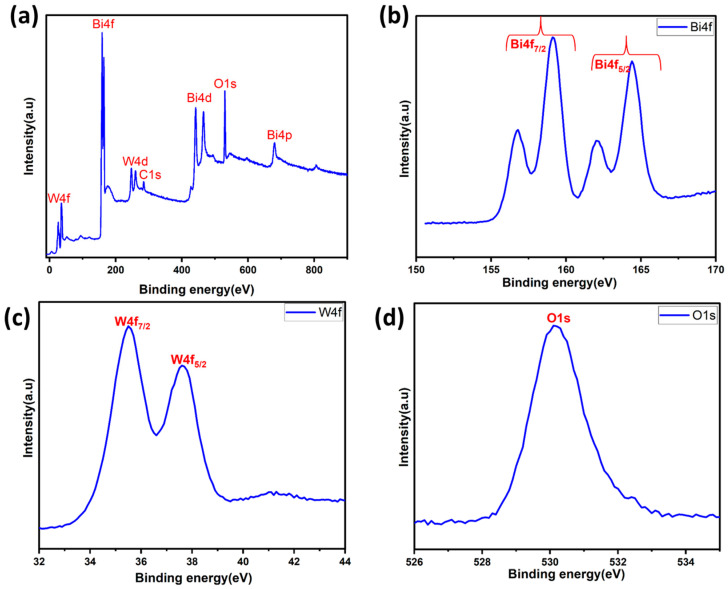
XPS survey (**a**) and high-resolution spectra of the BWO400 photocatalyst: Bi 4f (**b**), W 4f (**c**), and O 1s (**d**).

X-ray photoelectron spectroscopy (XPS) was also employed to determine the chemical states and surface chemical composition of the BWO400. Figure 5a presents the survey spectrum of the BWO400 material composed of W, Bi, and O elements and a little carbon trace. The existence of carbon can be explained by the use of a carbon tape within the XPS analysis [51]. Figure 5b shows the spin-orbit components of Bi 4f by two intense peaks found at 164.41 and 159.14 eV, assigned to the 4f_5/2_ and 4f_7/2_ states of the Bi^3+^ crystal structure, respectively [51]. Another spin-orbit doublet at 162.10 and 156.76 eV for Bi 4f_5/2_ and Bi 4f_5/2_ was also seen, respectively [52]. The peaks at 35.5 and 37.6 eV, as illustrated in Figure 5c, equivalent to W 4f_7/2_ and W 4f_5/2_, respectively, may be ascribed to a W^6+^ oxidation state [51,53,54]. Figure 5d reveals the existence of an oxygen element (O1s) via the clear, strong peak at 530.1 eV, which is assigned to the crystal lattice oxygen [55].

## 4. Assessment of the Photocatalytic Performance of BWO Photocatalyst

### 4.1. Adsorption and Photolysis Investigation

**Figure 6:**
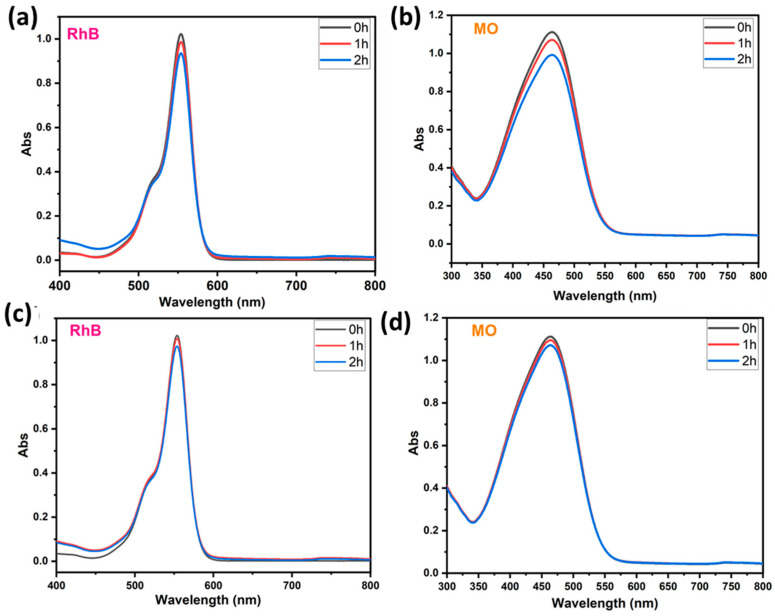
Visible absorption spectra of RhB and MO in the presence of BWO400 and in the absence of light (**a**,**b**); RhB and MO removal under illumination in the absence of BWO400 (**c**,**d**).

To prove the capability of our BWO400 material, it is important to study the adsorption of the pollutant in the absence of UV-vis illumination. Hence, the photolysis test enables the quantification of the photocatalytic decomposition contribution under our running parameters. Therefore, preliminary investigations were undertaken to check the contribution of the adsorption and the photolysis to the removal of the pollutant (case of RhB and MO). Figure 6a,b show the absorption curves of RhB (554 nm) and MO (463 nm) in the presence of BWO400 photocatalyst and in the absence of irradiation. It can be easily seen that the decline in the absorbance intensity of the highest RhB absorption bands is not more than 9% after 2 h of contact (Figure 6a), whereas in the MO it was 10.8% after 2 h of contact (Figure 6b). These results indicate very little adsorption of RhB and MO by the BWO400 material. The photolysis tests (absence of BWO400) were carried out on solutions (MO and RhB) with an initial dye concentration of 5 ppm under UV-vis illumination. Figure 6c,d reveal that in the absence of the BWO400 photocatalyst, only a reduction of 5% and 4% in the absorbance intensities was achieved after 2 h of illumination for RhB and MO, respectively. From these findings, we are able to confirm that the major process implicated in the removal of MO and RhB in the presence of BWO400 photocatalyst is principally photocatalytic decomposition instead of adsorption and photolysis processes.

### 4.2. Photocatalytic Activity and Kinetic Investigation of BWO

#### 4.2.1. The Case of Rhodamine B

**Figure 7:**
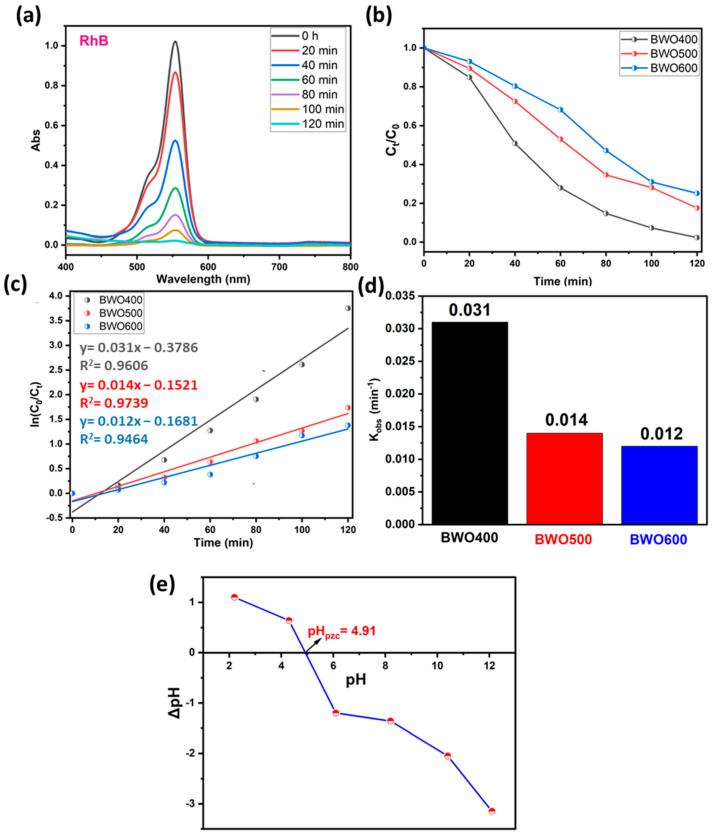
UV-vis absorption spectra of RhB under solar light illumination on BWO400 (**a**), the variation of *C_t_*/*C*_0_ with illumination time on BWO400, BWO500, and BWO600 (**b**), fitted pseudo-first-order kinetics for the photodecomposition reaction along with the corresponding rate constants (**c**,**d**), and the identification of point of zero charge (pH_pzc_) of BWO400 (**e**).

The photocatalytic performance of BWO treated at 400, 500, and 600 °C is assessed by the breakdwon of RhB dye molecules under light illumination. The photocatalytic dye decomposition efficiency curves of BWO calcined at several temperatures are illustrated in Figure 7a–d. For low concentrations (ppm), the Beer–Lambert law is applicable, so that the concentration is proportional to the absorbance. The photodecomposition efficacy was quantified by the ratios *C_t_*/*C*_0_ (Figure 7b), where *C_t_* and *C*_0_ are the concentrations of BWO at times *t* and *t* = 0, respectively. To evaluate the kinetic constant (Figure 7c,d), which describes the photocatalytic efficacy, a first-order kinetic rate law (Langmuir–Hinshelwood model) was used:

(5) $$ln\frac{c_{t}}{c_{0}}=-k_{obs}\cdot t$$

Figure 7a reveals that under UV illumination, the intensity of the contaminant absorption band declines with illumination time in the presence of BWO manufactured by co-precipitation method and heat-treated at 400 °C. Figure 7b illustrates the kinetics of photocatalytic decomposition in the presence of the different photocatalysts. More specifically, BWO calcined at 400 °C exhibited superior photocatalytic activity with 97% decomposition achieved within 120 min. This displays a better decomposition efficiency when compared to the BWO treated at 500 °C (82%) and 600 °C (75%). The results in Figure 7c demonstrate that the photocatalytic reaction obeys a pseudo-first-order kinetic rate law. The obtained rate constants *k_obs_* are given in Figure 7d. Their values are 0.031, 0.014, and 0.012 min^−1^ for the BWO400, BWO500, and BWO600 photocatalysts, respectively. The decline in photocatalytic activity when the calcination temperature increases is ascribed to the quick recombination of photo-induced electrons and holes. Moreover, the optical properties of BWO400, in particular its outstanding absorption of visible light and its low gap energy, encouraged us to adopt a lamp similar to solar illumination. In addition, this high efficiency of the BWO400 photocatalyst is also ascribed to the specific shape, featuring small, highly crystallized grains of the orthorhombic phase. It is worth mentioning that the as-produced material at this temperature presents good photocatalytic properties by delivering small particle size and more available surface sites for charge transfer [22,56]. These findings are consistent with XRD and SEM findings. To better understand and explain the mechanism behind the photocatalytic decomposition efficacy of RhB, we have measured the pH_pzc_ (Figure 7e). The pH_pzc_ is dependent on the acid-base characteristics of the surface material [25,57]. In this study, the orthorhombic BWO has a pH_pzc_ value equal to 4.91, and RhB is a cationic pollutant. At pH_i_ below or above pH_pzc_, the surface charge of BWO is negative or positive, respectively. This implies that the surfaces of BWO may be negatively or positively charged depending on the aqueous phase pH. In our system, the pH of our system was 5.5, which is greater than pH_pzc_, so BWO is negatively charged. As a result, the degradation efficiency is high owing to the electrostatic attraction occurring between the negatively charged catalyst surface and the positively charged cationic dye molecules. In addition, under alkaline media, photodegradation performance is more significant given the availability of hydroxyl ions, required for the creation of hydroxyl radicals.

#### 4.2.2. The Case of Methyl Orange

**Figure 8:**
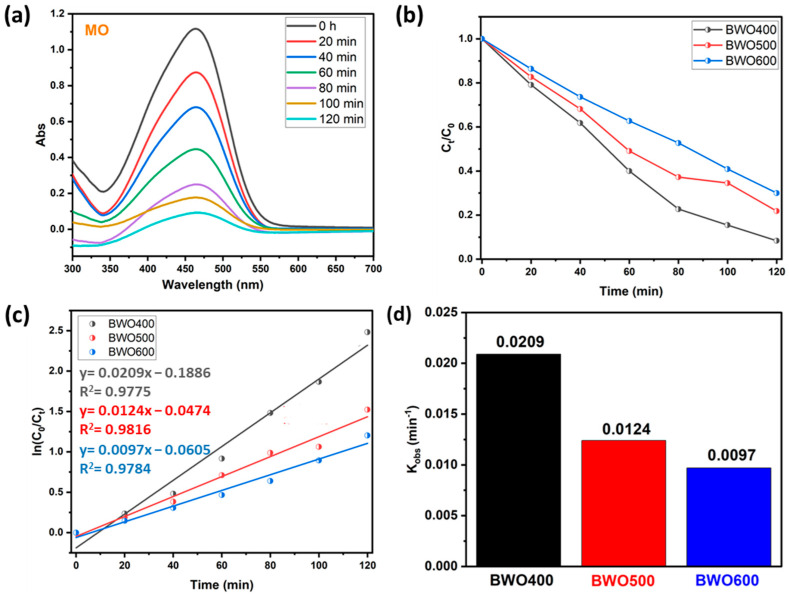
UV-vis absorption curve of MO under solar light illumination on BWO400 (**a**), the variation of *C_t_*/*C*_0_ with illumination time on BWO400, BWO500, and BWO600 (**b**), fitted pseudo-first-order kinetics for the photodecomposition (**c**), and corresponding rate constant of the photocatalytic reaction (**d**).

To ensure the photocatalytic performance of BWO prepared at various calcination temperatures, another experiment was carried out on the decomposition of an organic dye like methyl orange (anionic dye). Figure 8a reveals that under UV illumination, the intensity of the contaminant absorption band (465 nm) declines with illumination time in the presence of BWO as prepared by the co-precipitation method and thermally treated at 400 °C. In addition, Figure 8b shows plots describing the variation in *C_t_*/*C*_0_ of the MO versus illumination time for the different photocatalysts (BWO400, BWO500, and BWO600). Similar to RhB, the results obtained for MO show that the degradation efficiency decreases with the switch from BWO400 to BWO600. In particular, the BWO treated at 400 °C displayed superior photocatalytic performance, with a decomposition efficacy of 92% in 120 min, while the BWO500 presented an efficiency of 78%, and the decomposition efficiency for the BWO600 was 70% during 120 min of irradiation. Figure 8c indicates that the photocatalytic reaction obeys a pseudo-first-order kinetic. The determined rate constants *k_obs_* are given in Figure 8d. Their values are 0.0209, 0.0124, and 0.0097 min^−1^ for the BWO400, BWO500, and BWO600 photocatalysts, respectively. In addition to the optical characteristics, especially the gap energy and the PL readings, which show that BWO400 is more favorable for the degradation of MO, the achievement of this degradation efficiency (92%) can also be explained by the surface charge. In our reaction medium, the pH of our system is around 5.8, which is above pH_pzc_ (Figure 7e), so the BWO is negatively charged leading to a repulsion between the negatively charged MO molecules and the negatively charged BWO. This will result in a decrease in the pH of the medium toward a pH value lower than the pH_pzc_ and consequently the surface charge of BWO400 becomes positive. Consequently, the decomposing process is highly efficient owing to the electrostatic attraction occurring between the positively charged surface of the catalyst and the negatively charged dye molecule.

### 4.3. Investigation of the Photocatalytic Mechanism

**Figure 9:**
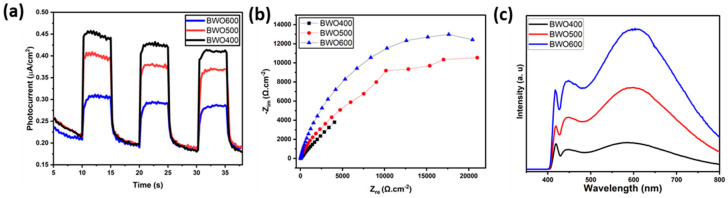
The transient photocurrent curves of BWO400, BWO500, and BWO600 (**a**), EIS Nyquist graphs (**b**) and the photoluminescence PL curves (**c**) of each photocatalyst (BWO400, BWO500, and BWO600).

The separation efficacy of the photo-induced charges is an essential parameter influencing the photocatalytic activity. To gain a better understanding of the mechanism of photocatalytic performance improvement, photoelectrochemical measurements and photoluminescence (PL) profiles were performed to characterize the separation behavior of the photo-induced charge. Figure 9a presents the transient photocurrent graphs of BWO400, BWO500, and BWO600. As shown, the photocurrent response of BWO400 is obviously greater than that of BWO500 and BWO600. The improved photocurrent density in BWO400 photocatalyst compared to BWO500 and BWO 600 is equivalent to a facilitated separation efficacy of the charge carriers, which greatly assists in improving the photocatalytic activity. It is interesting to note that the stable photocurrent density indicates good photoelectrochemical stability. In addition, Figure 9b displays the EIS Nyquist plots to examine the charge transfer characteristics of the as-prepared materials. It is widely known that the lower value of the arc radius is attributed to the quicker charge transfer rate and lower charge transfer resistance (R_ct_) [58,59,60,61]. It can be clearly seen that BWO400 exhibits a smaller arc radius when compared to BWO500 and BWO600, which indicates that BWO400 exhibits the quickest charge transfer rate and the highest effective separation efficacy, which is in close correlation with the transient photocurrent response findings. From the PL curve (Figure 9c) of the obtained photocatalysts, a higher PL intensity is clearly found in BWO600 in comparison with BWO500 and BWO400, which confirms that the enhanced transport and separation of the photo-generated charge is obtained by decreasing the calcination temperature. Furthermore, BWO400 displays the lowest PL intensity of all the prepared photocatalysts, reflecting the substantial increase in charge separation efficiency. Spectroscopic and photoelectrochemical investigations clearly reveal that the efficacy of photo-induced charge, migration, and separation is considerably boosted by lowering the heat treatment temperature. By reducing the temperature, damaging or denaturing the active sites may be avoided, allowing the photocatalyst to keep its catalytic activity. In addition, calcining at lower temperatures could lead to a band gap better adapted to the uptake of visible light. This enhanced absorption of light could improve photocatalytic activity [62].

### 4.4. Trapping Test

**Figure 10:**
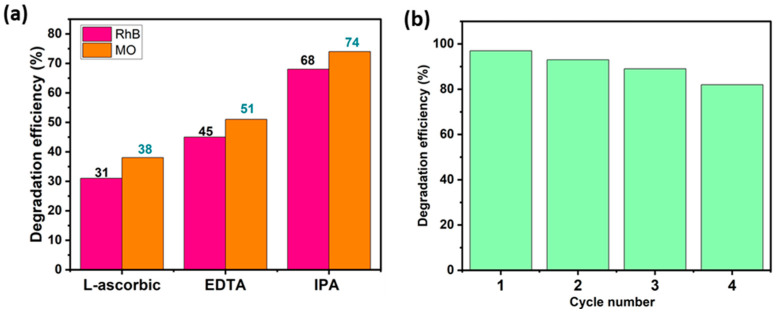
Photocatalytic decomposition efficacy of RhB and MO using BWO400 in the presence of a range of trapping agents (**a**), and the recycling test of the BWO400 photocatalyst (**b**).

Trapping studies were conducted out to identify the principal oxidizing species that are involved in dye decomposition. Ethylene-Diamine-Tetra-Acetic acid disodium (EDTA), L-ascorbic acid (L-asc), and Isopropyl Alcohol (IPA) were used as specific and appropriate scavengers of the species likely to be responsible for MO and RhB decomposition, i.e., OH^•^, O_2_^•−^ and holes h^+^. Figure 10a illustrates the impact of these trapping agents on the photocatalytic performance of the BWO400 photocatalyst with respect to RhB and MO. As can be seen in this figure, the photocatalytic breakdown efficiencies are 97% and 92% for RhB and MO, respectively, in the absence of scavengers. With the addition of L-ascorbic acid, EDTA, and IPA, these efficiencies dropped to 31%, 45%, and 68% for RhB and 38%, 51%, and 74% for MO, respectively. Therefore, it may be deduced at this stage that superoxide O_2_^•−^ and holes h^+^ are considered to be the principal species responsible for the photodecomposition of MO and RhB, while hydroxyl radicals OH^•^ are found to play a secondary function in the photocatalytic degradation. These results are similar to those of Wang et al. [63], who reported that O_2_^•−^ and h^+^ are the major species implicated in process decomposition.

### 4.5. Recyclability

The stability of the BWO400 catalyst was assessed by recycling the material after each photocatalytic reaction. The collected photocatalyst was rinsed and then dried for reuse. The experimental results presented in Figure 10b indicate that only a slight decline in photocatalytic activity was detected after four consecutive tests. This slight decrease could be due to photocatalyst loss during recycling.

### 4.6. Proposed Mechanism

**Figure 11:**
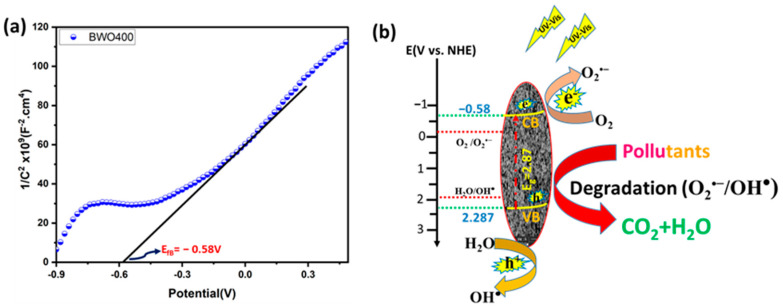
Mott–Schottky plot of the BWO400 photocatalyst (**a**), and the schematic band structure diagram depicting the proposed degradation mechanism (**b**).

To better explain the photocatalytic process, the valence band (VB) and the conduction band (CB) positions of the BWO400 were measured using Mott–Schottky (MS) analysis (Figure 11a). The MS formula is expressed below:

(6) $$C^{-2}=\frac{2}{N_{D}e\varepsilon \varepsilon_{0}}\left(V-V_{fb}-\frac{kT}{e}\right)$$

where *e*, *T*, *K*, *V_fb_*, *V*, *N_D_*, *ε*, *ε*_0_, and *C* were the electron charge, the absolute temperature, the Boltzmann constant, the flat band potential, the applied potential, the carrier density, the relative permittivity, the permittivity of free space and the space-charge capacitance, respectively. The corresponding curves show a linear variation of 1/*C*^2^ for greater potentials with a positive slope, which indicates that the prepared BWO400 is an n-type semiconductor (SC). The flat band potential (*V_fb_*) deduced from the x-intercept of the MS plots of BWO400 was −0.58 V vs. Ag/AgCl. Based on the recorded potential vs. Ag/AgCl, the normal hydrogen electrode (NHE) potential is calculated as E_Ag/AgCl_ = E_NHE_ − 0.197 [63,64]. Generally, the CB potential of n-type SC is more negative by approximately 0.2 V than its *V_fb_* [19,58,65]. The band structure of BWO400 photocatalyst can therefore be identified using the following equations:

(7) $$E_{CB}=V_{fb}-0.2$$

(8) $$E_{g}=E_{VB}-E_{CB}$$

Consequently, the value of *E_CB_* is found to be −0.78 V vs. Ag/AgCl, which is −0.583 V vs. NHE. The *E_VB_* is therefore obtained from the *E_CB_* and *E_g_* values and is equal to 2.287 V vs. NHE. To provide more insight about the active species of the BWO400 photocatalyst, we also display the potential of the couple O_2_ /O_2_^•−^ and H_2_O/OH^•^, which is −0.33 and 1.99 V vs. NHE, respectively [66]. As shown in Figure 11b, the potential of the VB of BWO is more positive than that of H_2_O/OH^•^, while the potential of the CB is more negative with respect to that of O_2_^•−^/O_2_. Therefore, according to the relative position of the band edges, the BWO400 photocatalyst may readily generate O_2_^•−^ and OH^•^ radicals [22,67]. Generally, the photodegradation of the dyes used involves an initial excitation of the Bi_2_WO_6_ semiconductor (Reaction (1)); excited electrons from the material can be transferred to the CB of the BWO semiconductor. These will react with the oxygen present in the solution to form the reactive species O_2_^•−^ (Reaction (2)). The holes in the valence band react with water (H_2_O) to form hydroxyl radicals OH^•^ (Reaction (3)). As a result, these reactive species formed (O_2_^•−^ and OH^•^) can effectively break down the organic pollutant into CO_2_ and H_2_O (Reaction (4)) [68]. Table 3 lists the photocatalytic efficiencies of BWO-based catalysts published in the literature, with various tested pollutants and illumination sources. In comparison with the reported photocatalyst in Table 3, the catalyst designed in this research exhibits a high photocatalytic activity compared to other BWO-based photocatalysts.

BWO + hv → BWO (e^−^, h^+^) (R1)

H_2_O + h^+^ → OH^•^ + H^+^ (R2)

O_2_ + e^−^ → O_2_^•−^ (R3)

(RhB, MO) + (OH^•^/O_2_^•−^) → CO_2_ + H_2_O (R4)

### 4.7. Estimation of RhB and MO Mineralization by COD Analysis

**Figure 12:**
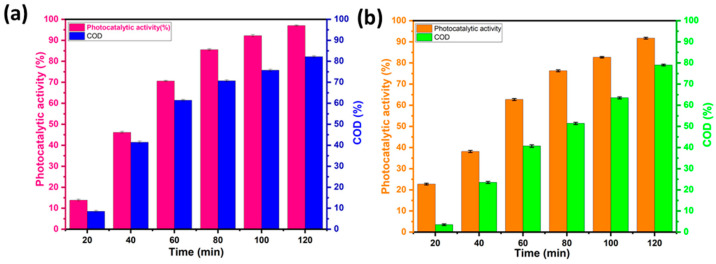
Variation in the chemical oxygen demand (*COD*) removal with the irradiation time for RhB (**a**) and MO (**b**).

The *COD* measurement provides an idea of the degree of mineralization of the contaminant after photocatalytic degradation and offers further information on the photocatalytic activity of the material. Figure 12a,b show the *COD* elimination rates when the BWO400 photocatalyst was used for the decomposition of RhB and MO. Figure 12 shows that as the irradiation time is increased, the *COD* reduction rate rises to 82% and 79% for RhB and MO, respectively, which indicates that mineralization of RhB and MO has occurred through the degradation of dye molecules into CO_2_ and H_2_O. The remarkable *COD* reductions observed in our study can be due to the specific characteristics of the BWO400 photocatalyst in terms of its morphology and optical and photoelectrochemical properties, whose mechanism of action relies on its capability to capture solar energy to activate catalytic reactions. The successful performance of BWO400 material in reducing *COD* levels demonstrates its potential as a viable material for water treatment applications.

## 5. Conclusions

In the present work, for environmental remediation, Bi_2_WO_6_ NPs (BWO) photocatalyst was designed by using a chemical precipitation method. Further, the effects of subsequent photocatalyst annealing at 400 °C, 500 °C, and 600 °C for organic dye photodegradation enhancement were investigated. It is shown that the photocatalyst calcination temperature has a profound influence on its morphology, its optical properties, its photoelectrochemical behavior, and its photocatalytic activity. The photocatalyst structural analysis revealed an orthorhombic phase, where the size of the uniform spherical grains increased with the calcination temperature. The BWO nanoparticles annealed at 400 °C demonstrated 97% and 92% elimination of RhB and MO model pollutants in photocatalytic degradation reactions after 2 h solar irradiation. In addition, the BWO annealed at 400 °C showed the highest transient photocurrent density among the studied catalysts along with a stable photoelectrochemical behavior and maintained its photocatalytic activity up to four cycles. The overall data indicate that the superoxide O_2_^•−^ and holes h^+^ are the major species responsible for the dye photodegradation.

## Figures and Tables

**Table 1:** Lattice parameters and volume of the unit cell of BWO NPs calcined at 400, 500, and 600 °C.

Treatment Temperature	*a* (Å)	*b* (Å)	*c* (Å)	Unit Cell Volume (Å)^3^
JCPDS card No. 01-079-2381	5.4373	16.4302	5.4584	487.6312
400 °C	5.4430	16.3134	5.4486	483.8021
500 °C	5.4428	16.3141	5.4474	483.6985
600 °C	5.4419	16.3204	5.4457	483.6543

**Table 2:** Frequency of the symmetric A_g_ modes of the terminal O–W–O stretching vibration and corresponding bond lengths for samples calcined at 400, 500, and 600 °C.

Treatment Temperature	Raman Stretching Frequency (cm^−1^)	Bond Length (Å)
400 °C	820	1.7003
500 °C	828	1.6952
600 °C	828	1.6852

**Table 3:** A comparative analysis of the photocatalytic efficacy of the BWO photocatalyst with other published photocatalysts.

Photocatalyst	Pollutants	Concentration (mg/L)	Light Source (W)	Efficiency (%)	Ref.
Sb^3+^/Bi_2_WO_6_	RhB and MO	6.5	Solar light	80.58 and 77.23	[69]
Bi_2_WO_6_/WO_3_	MO	-	Solar light	75.65	[70]
Bi_2_WO_6_	RhB	5	500 W Xe	95	[71]
Bi_2_WO_6_	RhB	5	500 W Xe	92	[72]
Bi_2_WO_6_/BiOCl	MO	10	Xe lamp	68	[73]
Fe-Bi_2_WO_6_	RhB	10	500 W Xe	93	[74]
Bi_2_WO_6_	RhB	4.8	300 W Xe	90	[75]
BWO400	RhB and MO	5	Philips lamps (300 W)	97 and 92	This study

## Data Availability

Data are contained within the article.
